# Establishment of the intracranial hemodynamic model based on contrast medium and clinical applications

**DOI:** 10.1097/MD.0000000000005550

**Published:** 2016-12-09

**Authors:** Yaoer Cheng, Wen He

**Affiliations:** Department of Radiology, Beijing Friendship Hospital, Capital Medical University, Beijing, China.

**Keywords:** CT perfusion, hemodynamic model, time-density curve

## Abstract

Ischemic cerebrovascular diseases are one of the most common vascular diseases in aged people and CT perfusion (CTP) is a very popular tool to detect the ischemic changes in brain vascular. The present study aims to establish a novel intracranial hemodynamic model to simulate anterior cerebral artery blood flow, and compare the actual and simulated hemodynamic parameters of healthy people and patients with carotid stenosis or occlusion.

A mathematical model of the intracranial hemodynamic was generated using MATLAB software, and data from patients with or without infarct disease (57 and 44 cases, respectively) were retrospectively collected to test the new model. The actual time-density curve (TDC) of anterior cerebral artery was obtained from the original intracranial CTP data, and simulated TDC was calculated from our intracranial hemodynamic model. All model parameters were adjusted according to patients’ sex, height, and weight. Time to peak enhancement (TTP), maximum enhancement (ME), and mean transit time (MTT) were selected to evaluate the status of hemodynamics.

In healthy people, there were no significant differences of TTP and ME between actual and simulated curves. For patients with infarct symptoms, ME was significantly decreased in actual data compared with simulated curve, while there was no obvious difference of TTP between actual and simulated data. Moreover, MTT was delayed in infarct patients compared with healthy people.

Our group generated a computer-based, physiologic model to simulate intracranial hemodynamics. The model successfully simulated anterior cerebral artery hemodynamics in normal healthy people and showed noncompliant ME and MTT in infarct patients, reflecting their abnormal cerebral hemodynamic status. The digital model is reliable and may help optimize the protocol of contrast medium enhancement in intracranial CT, and provide a solid tool to study intracranial hemodynamics.

## Abstract 2

Supplemental Digital Content is available in the text

## 1 Introduction

Ischemic cerebrovascular diseases are the most common cause of vascular dementia and death in aged people, and the internal carotid artery stenosis or occlusion is a common cause of stroke.^[1]^ Therefore, early diagnosis and intervention of ischemic cerebrovascular diseases is very important. To date, intracranial CT perfusion (CTP) imaging is the most valuable method for evaluating brain hemodynamics and cerebral vascular status. As a newly developed technique, CTP becomes favorable due to its wide range of applications, ability to quantitation, high image resolution and advantages of combining brain function and morphology.^[2]^ However, hemodynamic parameters of CTP can be easily affected by numerous factors including technical factors (species of contrast medium, injection speed, scan tube voltage, etc.) and patients’ individual differences (height, weight, cardiac output).^[3]^ Therefore, diagnosing cerebral diseases by the absolute values of perfusion parameters is quite difficult.

Brain CT angiography is a simple and noninvasive technique to show vascular lesions, occlusion, and plaque.^[4]^ The quality of CT angiography images depends largely on the density differences of cerebral vein and parenchymal. Therefore, CT scan delay time is very important since its influence on arterial CT enhancement.^[5]^ With standardized protocol of CTP (the uniformed contrast medium concentration, dosage, injection rate, and the scanning tube voltage, etc.), the circulation of contrast medium is attributable to patients’ individual characteristics, and accordingly the scan delay time has to be customized. Currently there are 3 common means to determine individual scan delay time, which are Test-Bolus,^[6]^ Bolus tracking^[7]^ and computer models to predict the distribution of the contrast medium.^[8]^ However, Test-Bolus technique is criticized by consuming additional contrast medium, increasing operational steps, and prolonging the inspection time.^[6]^ Bolus tracking^[9]^ technique increases radiation dose to patients, and is difficult to determine the threshold, and its carotid trigger failure rate is relatively high (36.67%)^[10]^ due to the small blood vessels in the neck, arterial pulse or involuntary movements and threshold selection. Recently, computer-based mathematical models have been taken seriously, for its reliability and convenience; it requires no additional contrast medium and customizes analytic parameters by patients’ gender, height, and weight.^[8]^ The math models serve to predict the concentration and distribution of contrast medium in different organs and simulate time-density curve (TDC) before clinical scanning to help doctors choose the best CT scan protocol.^[8]^ Bae et al^[8]^ demonstrated the first hemodynamic model of iodinated contrast medium in 1998. Since then, numerous theoretical models based on pharmacokinetics have been established. However, most models mainly applied in aorta and liver, while brain hemodynamics model still needs extensive study due to its complexity and broad application.

In the present study, our group employed MATLAB software to mathematically simulate iodinated contrast medium change in circulation and generated a reliable model to predict TDC curves. Further, we retrospectively collected over 100 patients’ CTP data to verify our dynamic model and compare key parameters in patients with or without cerebral infarction. To our best knowledge, we are the first to confirm the accuracy of computer-based intracranial hemodynamic model using large patients’ data. The study will provide a valuable methodology to clinical diagnosis and treatment.

## 2 Methods

### 2.1 Physiologically based pharmacokinetic and compartment models

To predict the concentration and distribution of the iodine-containing contrast medium in computer program, mathematical formulas, and models need to be generated to adequately reflect the actual situation of the simulation. Previous study has showed that the concentration of iodine-containing contrast medium in the artery or organs has a linear relationship with CT enhancement value.^[11,12]^ Therefore, CT value is a reliable reflection of the contrast medium's hemodynamics and blood distribution, which is the theoretical pillar of our study. The distribution of contrast medium depends on several body parameters, such as total body blood volume, cardiac output, various tissues and organs of blood volume, blood flow and overall fluid distribution,^[8]^ and all parameters were calculated according to previous studies.^[8]^ Compartment models constitute one of the basic steps of hemodynamic research. In the present study, vascular/cardiac central compartment model, organ model, and the overall circulatory system model from Bae et al^[8]^ were chosen to achieve the final cerebral hemodynamics model.

### 2.2 Model operation and simulation experiments

Forty-four calculus equations were generated to describe individual organ: macrovascular and the heart, respectively. Then, MATLAB software (MATLAB 7.0) was used to solve calculus equations to obtain TDC of the contrast medium in brain. Simulation experiments were fulfilled by inputting different body parameters to obtain different TDC.

### 2.3 Patients and CTP data

CTP data were collected retrospectively from patients performed CTP in Beijing Friendship Hospital, Capital Medical University between January 2008 to December 2012. Forty-four cases of patients were selected as healthy controls with no signs of cerebrovascular stenosis, cerebral perfusion abnormalities, or cerebral infarction, no medical record of heart and kidney diseases, including 19 males and 25 females. Fifty-seven patients with brain infarct were selected as diseased group, including 46 males and 11 females. Among those infarct patients, 42 patients showed lacunar infarction, 5 patients showed large artery infarction, and 10 patients showed combined lacunar cerebral infarction with large artery infarction. Most common site of the infarction was ventricle; others include basal ganglia, semi-oval center, frontal and parietal lobes, temporal parietal–occipital junction. Exclusion criteria are serious heart, liver, kidney disease. Age, sex, height, and weight of each patient were collected as demographic parameters. The study protocol was approved by the Research Ethics Committee of Beijing Friendship Hospital. All healthy people and infarct patients have signed the IRB consent before the CTA and CTP examinations.

Brain CTP was performed as standard protocol using GE light speed 64-slice spiral CT (USA). Nonionic contrast medium Ultravist (Bayer Ltd, Guangzhou, China) 45 mL was intravenously injected (flow rate of 4 mL/s, iodine contrast medium concentration 370 mg/mL), then scan after 5-second delay. Scan mode: tube voltage 80 kV, tube current 200 mA, scan time 50 s, thickness 5 mm, coverage 5 mm × 8 = 40 mm. All scans were operated by blinded radiologists independently. The perfusion raw data were loaded into GE Perfusion3 (CT perfusion 3, GE Healthcare; Fairfield City, Connecticut, USA) software to generate TDC. Time to peak enhancement (TTP) and maximum enhancement (ME) were recorded and mean transit time (MTT) was calculated by subtracting average peak times of venous and artery.

### 2.4 Simulated hemodynamic parameters

Body parameters of each patient (including gender, height, and weight) were input into pharmacokinetic model, and simulated TDC was obtained from MATLAB. Simulated TTP, ME, and MTT were calculated from the digital model.

### 2.5 Statistics analysis

All values are expressed in the form of mean ± SEM. Paired *t* test was used to compare actual and simulated parameters. *P* values below 0.05 were considered as statistically significant. All statistical analysis was completed by the SPSS 17.0 software.

## 3 Results

### 3.1 Intracranial hemodynamic model was generated and tested using real patients’ body parameters

**Table:**
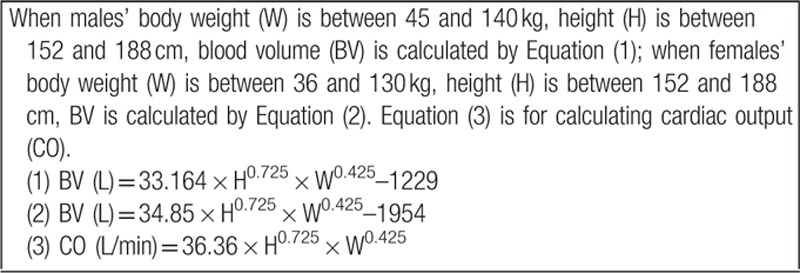

**Figure 1:**
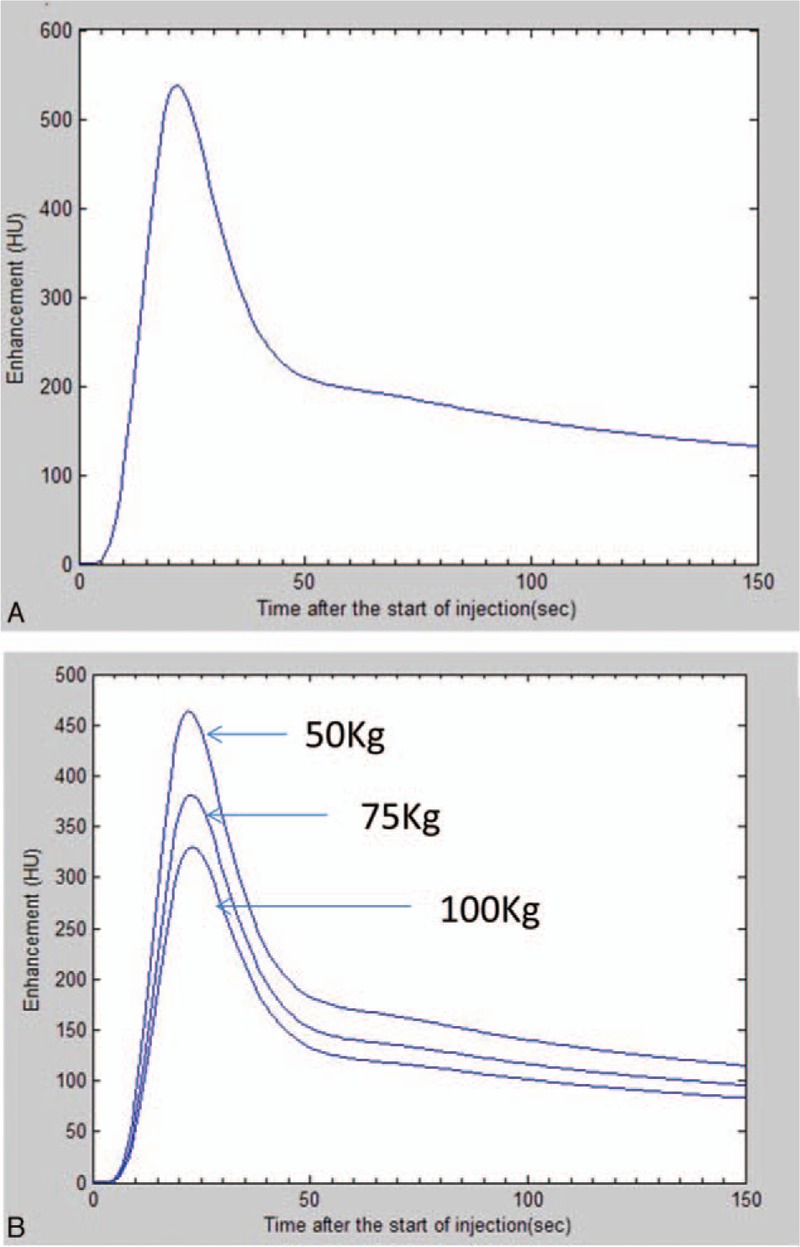
Simulated TDC. Personalized body parameters were input into hemodynamic model and simulated TDCs were generated from the computer. (A) A simulated TDC of normal male adult with a height of 170 cm and a weight of 75 kg; (B): 3 TDC with body weight of 50, 75, and 100 kg.

Computer-based hemodynamic model was generated using MATLAB. In this model, cardiac output of all patients is assumed as 100%. Therefore, blood volume (BV) and blood flow to various organs of each patient can be calculated. Hypothetically, if the patient uses the standard protocol for CT scan, concentration of iodine contrast medium is 370 mg/mL; injection rate is 4 mL/s, the total amount of contrast medium is 45 mL, and the tube voltage is 80 kV. Given the patient's height (H) and weight (W), TDC of anterior cerebral artery can be drawn by computer. Figure 1A shows a simulated TDC of normal male adult with a height of 170 cm and a weight of 75 kg; and Fig. 1B shows 3 TDC with body weight of 50, 75, and 100 kg, demonstrating that under the same condition and same height, the body weight affects simulated TDC which is similar to actual patients.

### 3.2 Patient characteristics

Forty-four cases of healthy controls and 57 patients with brain infarct showed no significant differences in age and body weight, however, there were more males in infarct group while more females in healthy group (Table 1). Since the gender ratio is not equivalent between 2 groups (*P* < 0.005 based on Chi-square test), the present study compared the experimental parameters: ME, TTP, and MTT by gender. The results showed significant differences of ME and MTT between men and women in control group, but no significant difference of all 3 parameters in infarct group (Supplemental Table 1). Healthy control population include people with no signs of cerebrovascular stenosis, cerebral perfusion abnormalities or cerebral infarction, no medical record of heart and kidney diseases, and patients with brain infarct include unilateral or bilateral carotid artery stenosis or occlusion.

**Table 1:**
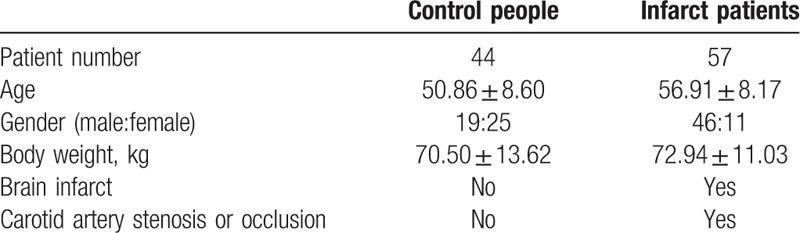
Patient characteristics.

### 3.3 Comparison of actual and simulated ME and TTP in 44 healthy controls

The actual TDC and hemodynamic parameters were obtained from retrospective CTP data. In 44 patients without any cardiovascular diseases, the average ME was 409.96 Hu. Average male ME was 365.47 Hu, and the average female ME was 443.77 Hu, indicating a higher ME in female (Table 2). The average TTP indicating contrast medium to anterior cerebral artery was 21.41 seconds, while in male it was 22.14 seconds, and in female it was 20.85 seconds (Table 2). The simulated TDC was calculated from our digital model. The average ME was 416.34 Hu, and the average TTP was 22.13 seconds (Table 2). There were no significant differences between the actual and simulated ME, nor between the actual and simulated TTP, demonstrating that our hemodynamic model succeeded in simulating the concentration and distribution of contrast medium in human bodies.

**Table 2:**
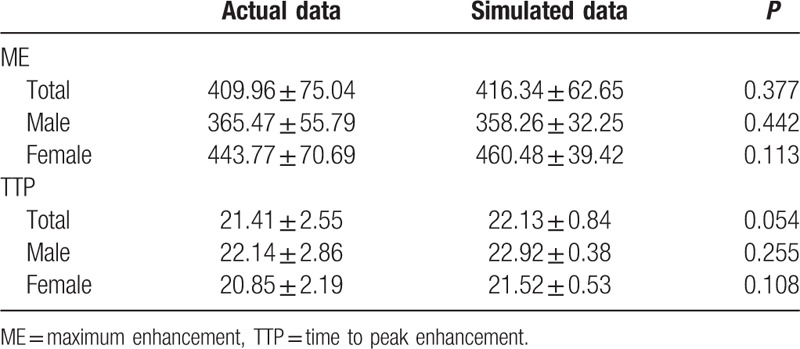
Comparison of actual and simulated ME and TTP in 44 healthy controls.

**Figure 2:**
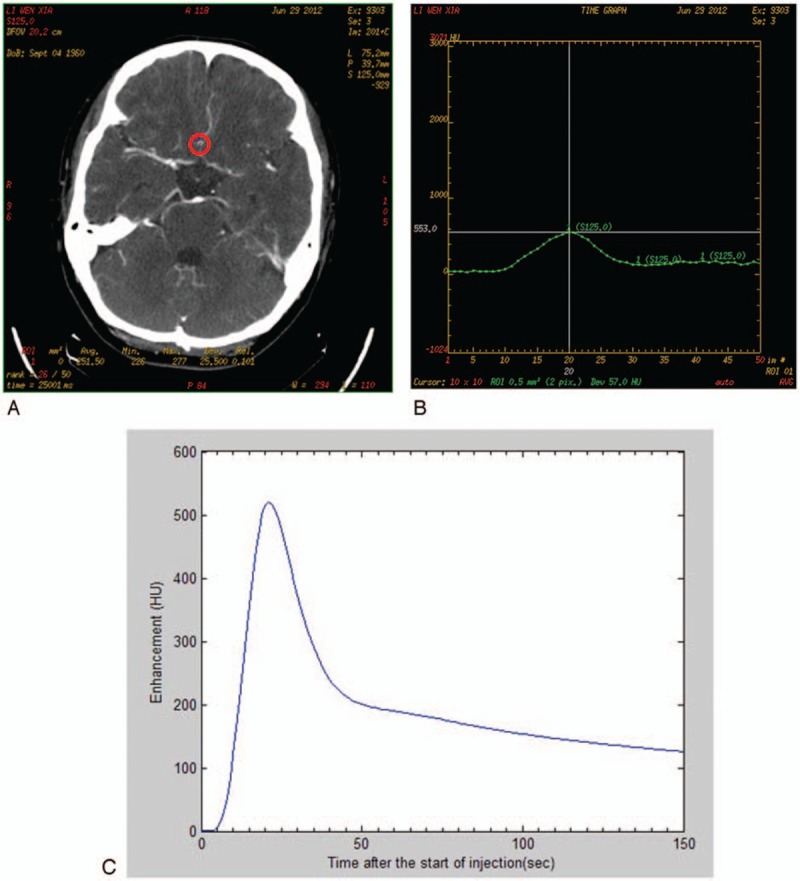
One example of actual TDC and simulated TDC. (A) Region of interest for CTP. Red circle in the CT image refers to region of interest. (B) Actual TDC of 1 healthy person. (C) Simulated TDC of the same person.

Figure 2 represents an example of a real patient. Region of interest was selected in the anterior cerebral artery indicated by the red circle in Fig. 2A. The actual TDC showed that the peak value was 553 Hu, and the peak time (TTP) was 20 s. After 3 measurements, the patient's actual peak value was 558.6 Hu, subtracting the background value 50 Hu, thus the actual ME was 508.6 Hu, the actual peak average of 20 seconds (Fig. 2B). The simulated TDC of the same patient is shown in Fig. 2C, simulated ME was 520 Hu, and simulated TTP was 21 seconds. There were no differences between the actual and simulated ME/TTP.

### 3.4 Comparison of actual and simulated ME and TTP in 57 infarct patients

Similarly, the actual TDC and hemodynamic parameters were obtained from retrospective CTP. The average actual ME was 337.81 Hu, and the average TTP was 21.33 seconds. The simulated ME was 391.04 Hu, and average TTP was 22.32 seconds. Paired *t* test showed that, there was significant difference between the actual and simulated ME in infarct patients, while no statistical difference between the actual and simulated TTP (Table 3).

**Table 3:**
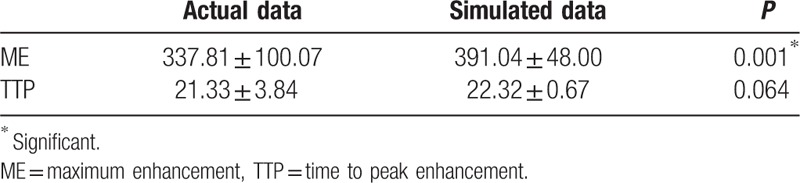
Comparison of actual and simulated ME and TTP in 57 infarct patients.

### 3.5 Comparison of ME, TTP, and MTT between infarct patients and healthy controls

Since the gender ratio is not equivalent between healthy control group and infarct patient group, we compared the parameters between 2 groups in different gender. In men, there was no difference of ME, TTP, or MTT between control and infarct patients, while in women, only MTT showed significant difference between control and infarct patients (Table 4). MTT was measured as the average amount of time it takes for the blood to transit through the given volume of brain. For women with infarction, MTT was significantly delayed compared with control group.

**Table 4:**
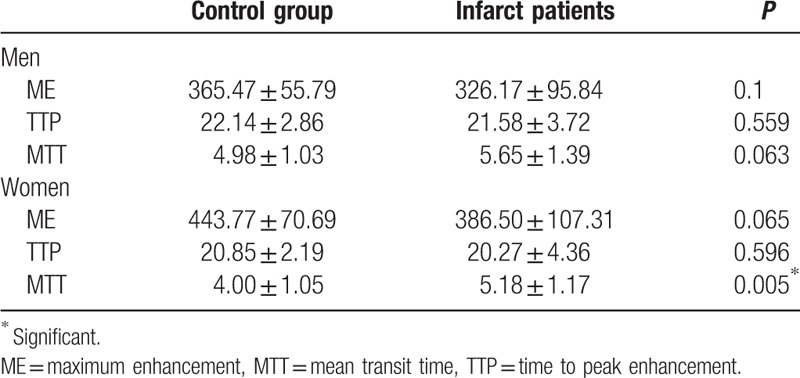
Comparison of ME, TTP and MTT between infarct patients and healthy controls.

## 4 Discussion

Spiral CT scan becomes more and more important in diagnosing brain vascular diseases. There are many factors that can influence the contrast medium-enhanced effects, such as the type, dosage, injection rate of the contrast medium, and the patient's body parameters. Currently, there are several techniques to predict the arterial peak enhancement plateau to achieve better reinforcing effect, including Test-Bolus and Bolus tracking.^[6]^ However, consuming extra contrast medium or increasing additional radiation to patients makes those techniques less appealing. To date, the biggest challenge in clinical application of CTP is the radiation dose. Traditionally, perfusion scan requires continuous scanning for about 50 seconds; some recent studies suggest interval scanning with interval time less than 3 seconds.^[13]^ However, the exposure dose is still quite large. On the other hand perfusion parameters are affected by body weight and cardiac output, the appropriate method to calculate and correct this problem still needs exploring. Modern studies are focused on establishing a model of the human cardiovascular disease based on the development of computer technology.^[8]^

The present study employed the method of derivation to model the cardiovascular system, which refers to construct a mathematical system to simulate the mechanism and function of the original system. Further, after computer calculation, the experts have been able to predict the change of contrast medium over time in vitro. To date, there have been various digital models to predict TDC, such as Bae physiological pharmacokinetic model,^[8]^ Uma Fourier transform method,^[14]^ Corey separated pharmacokinetic model,^[15]^ biomechanics method,^[16]^ and Upton first cycle model.^[17]^ Our group chose Bae physiological pharmacokinetic model to generate our hemodynamic model due to its application in human cardiovascular circulatory system and simplicity to implement. We simulated the circulation of iodinated contrast medium in brain perfusion, from the superior vena cava into the left heart, through pulmonary circulation to reach the aorta, then the process to reach the brain from the circulation. We also simplified the pharmacokinetic model to make it easier to use, through omitting the impact of contrast medium refluxing to the venous. Our study proved that, hemodynamics parameters were changed in patients with internal carotid artery stenosis compared with healthy people or simulation of normal standard values.

The present study collected nearly 100 cases with or without brain infarct from CTP data to test and confirm our mathematic model. There are no statistical differences between the actual and simulated TDC in healthy population (average ME difference of 10.29% and the average TTP difference of 9.77%), indicating that the model is quite reliable to predict the intracranial distribution and concentration of the contrast medium. The computer model provides a visual and quantitative method to study cerebral hemodynamic changes under different sign parameters. It provides important information for selecting scan program in CTP examination, such as adjusting the delay time of contrast medium injection and the amount of contrast medium to CT enhance scan by patient's gender, height, and weight.

The most popular application of our model would be in diagnosing carotid artery stenosis, which is a very common disease in aged population. In the United States, 25% ischemic stroke patients suffer from carotid stenosis lesions.^[18,19]^ North American Symptomatic Carotid Endarterectomy Trial (NASCET) showed that 20% to 30% of ischemic cerebrovascular diseases are secondary to internal carotid artery stenosis.^[20]^ Therefore, early detection of head and neck stenosis and cerebral perfusion is very important to provide further prevention and treatment of ischemic stroke. Atherosclerosis is the main cause of carotid artery stenosis,^[21]^ which not only causes luminal stenosis, but also results in hemodynamic changes, including carotid laminar flow disappear and blood flow disorder. Under normal condition, cerebral blood vessels have self-regulation (cerebrovascular reserve capacity)^[22]^ to maintain cerebral blood flow within a relatively stable range. However, vascular stenosis leads to blood flow reduction and inadequate compensation of collateral circulation, which leads to reduced cerebral perfusion pressure. Low cerebral perfusion pressure performances decreased cerebral blood flow and volume, which indicates prolonged MTT and TTP. Prolonged TTP indicates slower blood flow or inadequate collateral circulation compensation,^[23]^ and Grandin et al^[24]^ think that MTT is an indicator of cerebral perfusion pressure, which objectively reflects the time of cerebral blood microcirculation. Therefore, MTT and TTP are considered to be sensitive indicators of brain perfusion injury, especially for early detection of cerebral ischemic changes.^[25]^ Several studies showed that MTT and TTP were extended even in well-compensated artery stenosis patients with good collateral cerebrovascular, when there were no obvious changes in cerebral blood flow and volume.^[26]^

In this study, we compared TTP and ME between infarct patients with normal simulated TDC. Our data showed that there were no significant differences in TTP, but ME was increased in simulated model. Since the present study measured the TDC of ipsilateral cerebral artery, TTP in this study only represents the peak time of medium at the anterior cerebral artery, rather than the peak time in local brain tissue, therefore, TTP may be extended in local brain tissue, but not at anterior cerebral artery if there is no stenosis. Additionally, there are numerous factors outside of the brain to affect TTP, such as cardiac output, aortic plaque, and individual differences, etc. Consequently, variation of TTP in this study was huge (9–33 s), which explains the comparable TTP between infarct and healthy people. In contrast, since blood flow of anterior cerebral artery was reduced in infarct patients, MTT was prolonged for females compared with healthy control.

However, there are several limits of this study. The model does not simulate venous TDC in the brain; therefore, we cannot obtain personalized MTT value. We had to compare control population's actual MTT with infarct patients’ actual MTT, which could be affected by certain personal characteristics. Plus there were fewer males in the study, so MTT values in males only showed a trend of increase yet not any significant difference between healthy people and infarct patients. Besides, this study did not grade infarct patients by different degrees of stenosis, and did not consider intracranial vascular stenosis, Willis ring integrity, collateral compensation of arterial circulation and other issues, which could largely affect hemodynamic parameters. Nevertheless, this is a pilot study to show using mathematic model to predict TDC and compare with real patients’ data. The study provides a potential usage of mathematical models to predict the change of contrast medium circulation, which could reduce the radiation dose, and redress body weight, cardiac output, and other factors affecting data. We believe this model will definitely benefit patients in the future.

## Supplementary Material

Supplemental Digital Content
