# Supplementary material for: Establishment of the intracranial hemodynamic model based on contrast medium and clinical applications
Source: Medicine (Baltimore). 2016 Dec 9;95(49):e5550. doi: 10.1097/MD.0000000000005550 (PMC5266027; doi:10.1097/MD.0000000000005550)
Supplement: Supplemental Digital Content [file medi-95-e5550-s001.docx]

**Supplemental Table 1. Comparison of ME, TTP and MTT between Men and Women within group.**

|  | | **Men** | **Women** | **P value** |
| --- | --- | --- | --- | --- |
| **Control Group** | ME | 365.47±55.79 | 443.77±70.69 | <0.0001 |
|  | TTP | 22.14±2.86 | 20.85±2.19 | 0.098 |
|  | MTT | 4.98±1.03 | 4.00±1.05 | 0.003 |
| **Infarct Patients** | ME | 326.17±95.84 | 386.50±107.31 | 0.072 |
|  | TTP | 21.58±3.72 | 20.27±4.36 | 0.315 |
|  | MTT | 5.65±1.39 | 5.18±1.17 | 0.303 |
